# Increased Mucosal IL-22 Production of an *IL-10RA* Mutation Patient Following Anakinra Treatment Suggests Further Mechanism for Mucosal Healing

**DOI:** 10.1007/s10875-016-0365-3

**Published:** 2017-01-07

**Authors:** Jian Li, Dror S. Shouval, Andria L. Doty, Scott B. Snapper, Sarah C. Glover

**Affiliations:** 1 Division of Gastroenterology, Hepatology and Nutrition, Department of Medicine, University of Florida, 1600 SW Archer Rd, PO Box 100214, Gainesville, FL 32610 USA; 2 Division of Pediatric Gastroenterology and Nutrition, Edmond and Lily Safra Children’s Hospital, Sheba Medical Center, 52621 Tel Hashomer, Israel; 3 Sackler Faculty of Medicine, Tel Aviv University, 6997801 Tel Aviv, Israel; 4 Division of Gastroenterology, Hepatology and Nutrition, Boston Children’s Hospital, Boston, MA 02115 USA; 5 Division of Gastroenterology, Brigham and Women’s Hospital, Boston, MA 02115 USA; 6 Department of Medicine, Harvard Medical School, Boston, MA 02115 USA

To the Editor:

IL10 is an immunoregulatory cytokine that has a central role in the maintenance of intestinal mucosal homeostasis and prevention of colitis [1]. Loss-of-function mutations in *IL10*, *IL10RA*, or *IL10RB* cause severe intestinal inflammation and perianal disease that presents in the first months of life and is refractory to conventional immunosuppressive medications such as steroids, anti-TNFα antibodies, and immunomodulators [2]. The definitive treatment for IL10R deficiency is an allogeneic hematopoietic stem cell transplantation (HSCT) to reconstitute IL10 signaling, if a suitable donor is available. Unfortunately, the clinical condition of many of these patients is not amenable to transplant at the time of their diagnosis. As such, a therapeutic bridge is needed to prepare them for transplant.

We have recently shown that IL1β is highly upregulated in IL10R deficiency [3], and blocking IL1 attenuated colitis in an IL10R-deficient mouse model. Thus, we elected to treat two patients with medical-refractory severe inflammatory bowel disease (IBD) secondary to IL10R deficiency with anakinra, an IL1 receptor antagonist. In both cases, high dose anakinra therapy (10–12 mg/kg/day) led to marked clinical, endoscopic, and histological improvement within a few weeks [3]. To date, this is the first report of treatment that suppressed the hyperactive immune response in the gut of these patients.

Herein, we present additional information on one of these patients, a 28-year-old IL10R-deficient patient with a history of severe infantile-onset IBD, whose condition improved significantly after anakinra treatment. This patient initially presented with bloody diarrhea and was diagnosed with IBD at 6 months of age. He underwent a subtotal colectomy with end ileostomy at age 7 years and developed enterocutaneous fistulae at the age of 18. At age 23, a large B cell lymphoma was discovered and he received R-CHOP chemotherapy. An ileoscopy at age 28 demonstrated severe ileitis (Fig. 1a) and examination of the abdominal wall revealed multiple enterocutaneous fistulae. After the confirmation of an *IL10RA* loss-of-function mutation in this patient, he was started on anakinra. During the initial 2 weeks of treatment dose escalated to 12 mg/kg/day subcutaneously. At 4 weeks, a repeat ileoscopy showed he had a substantial improvement in his mucosal inflammation (Fig. 1a). The patient also experienced a significant decrease in his ileostomy output and marked weight gain within a few months. There were no significant changes in his serum inflammatory markers. Overall, the patient was treated with anakinra for 6 months without any significant side effects and is currently being prepared for an allogenic HSCT [3].

**Fig. 1:**
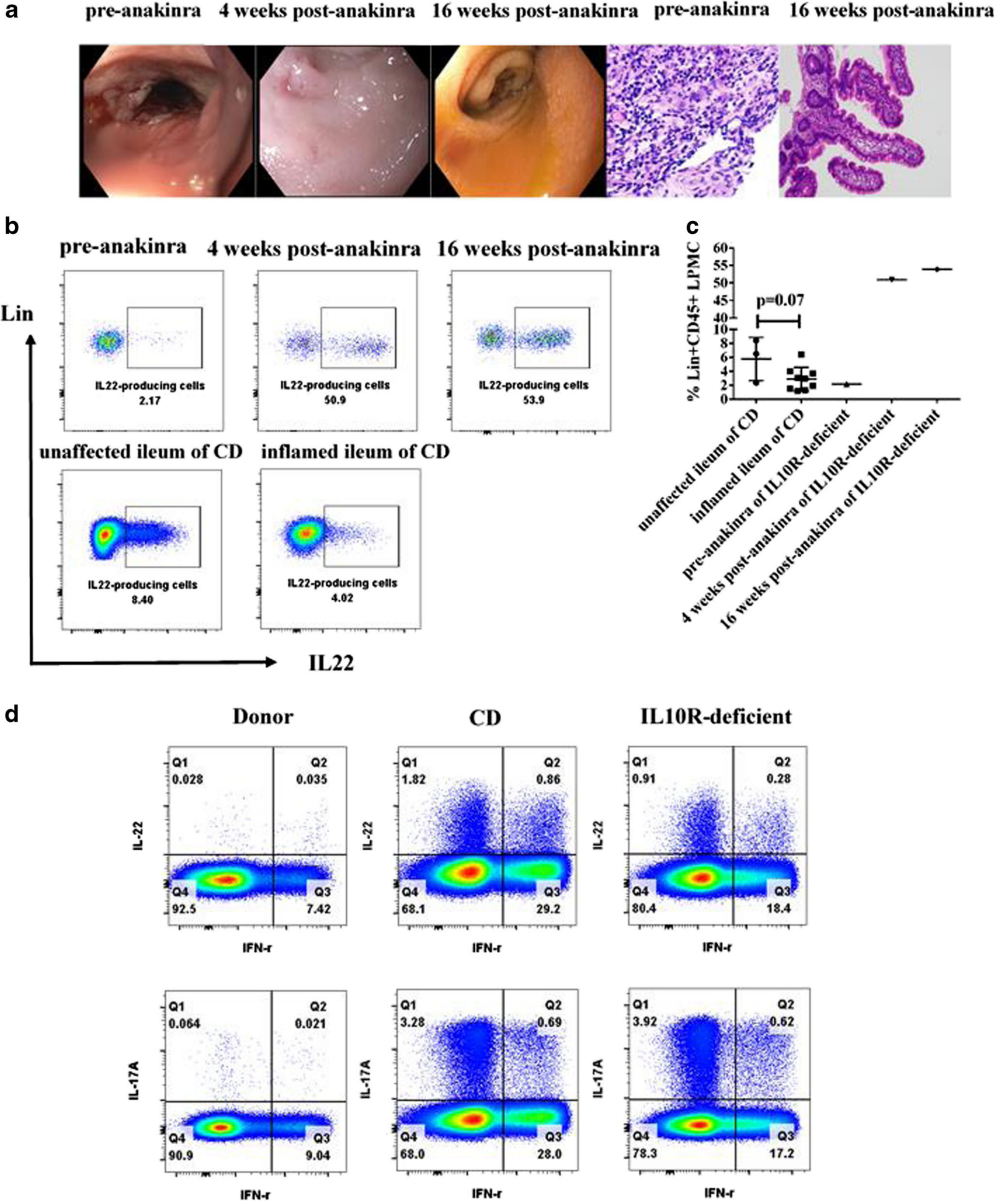
Anakinra treatment induces increased IL22 production in the terminal ileum and peripheral blood of IL10R-deficient patient

To further understand the mechanism of anakinra treatment in the IL10R-deficient patient, we compared the frequency of IL22-producing lymphocytes in lamina propria mononuclear cells (LPMC) isolated from his terminal ileum biopsy samples taken pre- and post-anakinra treatment. IL22-producing lymphocytes were our primary targets because of their known role in healing of intestinal inflammation. In the lineage negative compartment, we did not find IL22-producing innate lymphoid cell group 3 (ILC3). However, the frequency of IL22-producing lymphocytes among lineage (+) CD45 (+) LPMC greatly increased from 2.17% pre-anakinra to 50.9% post-anakinra (Fig. 1b). Analysis of an additional ileal biopsy 3 months later demonstrated that the number further increased to 53.9% among lineage (+) CD45 (+) LPMC (Fig. 1b). Furthermore, H&E staining of this additional biopsy showed resolution of the severely inflamed ileal region 5 cm above the ileostomy (Fig. 1a). The remaining 5 cm with associated enterocutaous fistulae remained inflamed. In addition, as mentioned in our recent publication, the inflammatory changes in the skin adjacent to the patient’s ileostomy and fistulae also resolved. Overall, our data suggest that anakinra-induced IL22 production may have a role in mucosal healing in this IL10R-deficient patient.

During a recent clinical trial among hidradenitis suppurativa patients, Vassiliki et al. observed significantly increased production of IL22 by peripheral blood mononuclear cells (PBMC) following anakinra therapy and concluded that the increased IL22 might contribute to an improved epithelial cell defense [4]. Based on these observations, we decided to assess IL22 production in the LPMC compartment of our sporadic adult Crohn’s disease population (Supplementary Table. 1). We detected a trend towards reduction of the IL22-producing lymphocytes (*p* = 0.07) among Lineage (+) CD45 (+) LPMC in the inflamed tissue (2.89%, *n* = 9) compared to unaffected tissue (5.75%, *n* = 3) (Fig. 1c). This result complements another report that IL22-producing CD4+ cells were depleted in active inflamed lesions of ulcerative colitis (UC) patients [5].

The deficiency of IL22-producing lymphocytes in the inflamed gut and the fact that its recovery coincided with improved intestinal inflammation suggest a protective role of this population in the gastrointestinal tract and that it might serve as a surrogate marker of mucosal healing. IL22 is a member of the IL10 family of cytokines. It directly binds to the heterodimeric receptor IL22Rα1-IL10Rβ on non-hematopoietic cells, especially on epithelial cells. The binding of IL22 promotes epithelial cell proliferation; secretion of antimicrobial peptides such as RegIIIβ, RegIIIγ, S108a, and S109a; and mucus production as well as fucosylation. Specifically, IL22 stimulation leads to the activation of signal transducer and activator of transcription 3 (STAT3) in epithelial cells, which contributes to tissue repair after injury. Finally, it is important to mention that this patient is an *IL10RA* mutation and his *IL10RB* is intact. This guarantees the responsiveness of epithelial cell to the increased IL22 signaling. Response to anakinra in IL10RB-deficient patients might be less effective but warrants further evaluation.

We subsequently assessed whether the frequency of circulating IL22-producing lymphocytes was altered in our IL10R-deficient patient, when compared to a Crohn’s disease cohort and normal controls. Indeed, we observed a significant increase in blood-borne TH22 and TH17 cells, both single producers and IL22/IFNγ, IL17A/IFNγ double producers, in the patient, as well as in Crohn’s disease subjects (Fig. 1d and Supplementary Fig. 1). It is important to note that the IL10R-deficient patient was analyzed after starting anakinra therapy, which we know decreased mucosal IL17 in both treated patients and might also influence circulating TH17 cells [3].

In conclusion, we present a case of a patient with severe IBD due to an *IL10RA* mutation who demonstrated a dramatic response to anakinra therapy. This intervention led to mucosal healing accompanied by increased frequency of IL22-producing lymphocytes in the lamina propria of the terminal ileum. The increase in IL22, which promoted tissue repair, might have had an important role in the recovery of this patient and suggests an additional important mechanistic mode of action of anakinra in the gut.

## Electronic Supplementary Material

Supplementary Table 1 The clinical characteristics of patients with CD. (DOCX 11 kb)

Supplementary Figure 1 Quantification of the TH17 and TH1 cytokine-producing lymphocytes among CD45(+) Lineage (+) or CD45(+) CD3(+) PBMC among normal control patients (*n* = 5), CD patients (*n* = 7) and the IL10R-deficient patient. (DOCX 90 kb)

Supplementary Figure 2 Gating strategy for the IL22-producig lymphocytes identification. (DOCX 62 kb)

## References

[1] Shouval DS, Ouahed J, Biswas A, Goettel JA, Horwitz BH, Klein C (2014). Interleukin 10 receptor signaling: master regulator of intestinal mucosal homeostasis in mice and humans. Adv Immunol.

[2] Glocker EO, Kotlarz D, Boztug K, Gertz EM, Schaffer AA, Noyan F (2009). Inflammatory bowel disease and mutations affecting the interleukin-10 receptor. N Engl J Med.

[3] Shouval DS, Biswas A, Kang YH, Griffith AE, Konnikova L, Mascanfroni ID et al. Interleukin 1 beta mediates intestinal inflammation in mice and patients with IL10 receptor deficiency. Gastroenterology. 2016. 10.1053/j.gastro.2016.08.055 PMC5124405 27693323

[4] Tzanetakou V, Kanni T, Giatrakou S, Katoulis A, Papadavid E, Netea MG (2016). Safety and efficacy of anakinra in severe hidradenitis suppurativa: a randomized clinical trial. JAMA Dermatol.

[5] Leung JM, Davenport M, Wolff MJ, Wiens KE, Abidi WM, Poles MA (2014). IL-22-producing CD4+ cells are depleted in actively inflamed colitis tissue. Mucosal Immunol.

