# Supplementary material for: Increased Mucosal IL-22 Production of an IL-10RA Mutation Patient Following Anakinra Treatment Suggests Further Mechanism for Mucosal Healing
Source: J Clin Immunol. 2017 Jan 7;37(2):104–7. doi: 10.1007/s10875-016-0365-3 (PMC5325838; doi:10.1007/s10875-016-0365-3)
Supplement: Supplementary file 1 — The clinical characteristics of patients with CD. (DOCX 11 kb) [file 10875_2016_365_MOESM1_ESM.docx]

**Supplementary Table. 1. The clinical characteristics of patients with CD.**

| CD patients | Gender | Age | Medication |
| --- | --- | --- | --- |
| Patient 1 | M | 24 | Anti-TNFα Humira |
| Patient 2 | M | 58 | Anti-TNFα Remicade |
| Patient 3 | M | 28 | Anti-TNFα Remicade |
| Patient 4 | M | 25 | Anti-IL-12/23 p40 Ustekinumab |
| Patient 5 | M | 34 | Mesalamine |
| Patient 6 | F | 57 | Anti-α4β7 Entyvio |
| Patient 7 | M | 24 | Anti-TNFα Humira |
| Patient 8 | F | 35 | Anti-IL-12/23 p40 Ustekinumab |
| Patient 9 | F | 34 | Anti-α4β7 Entyvio |
| Patient 10 | F | 60 | Anti-α4β7 Entyvio |
| Patient 11 | F | 40 | Anti-TNFα Remicade |
| Patient 12 | F | 36 | Anti-IL-12/23 p40 Ustekinumab |
| Patient 13 | M | 37 | Anti-IL-12/23 p40 Ustekinumab |
