# Supplementary material for: Increased Mucosal IL-22 Production of an IL-10RA Mutation Patient Following Anakinra Treatment Suggests Further Mechanism for Mucosal Healing
Source: J Clin Immunol. 2017 Jan 7;37(2):104–7. doi: 10.1007/s10875-016-0365-3 (PMC5325838; doi:10.1007/s10875-016-0365-3)

**Supplementary Figure 1: Quantification of the TH17 and TH1 cytokine-producing lymphocytes among CD45(+) Lineage (+) or CD45(+) CD3(+) PBMC among normal control patients (n=5), CD patients(n=7) and the IL10R-deficient patient.**


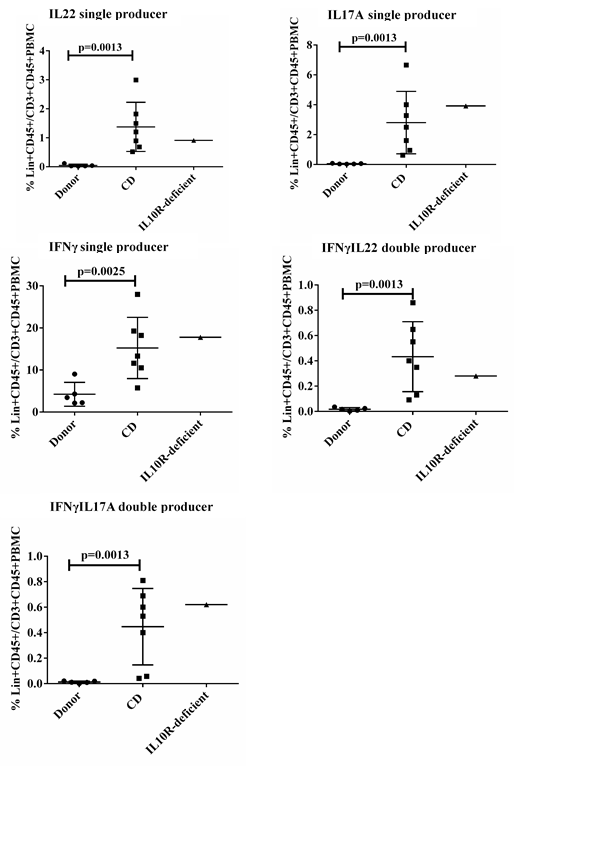

Supplement: Supplementary file 2 — Quantification of the TH17 and TH1 cytokine-producing lymphocytes among CD45(+) Lineage (+) or CD45(+) CD3(+) PBMC among normal control patients (n = 5), CD patients (n = 7) and the IL10R-deficient patient. (DOCX 90 kb) [file 10875_2016_365_MOESM2_ESM.docx]
