# Supplementary material for: Increased Mucosal IL-22 Production of an IL-10RA Mutation Patient Following Anakinra Treatment Suggests Further Mechanism for Mucosal Healing
Source: J Clin Immunol. 2017 Jan 7;37(2):104–7. doi: 10.1007/s10875-016-0365-3 (PMC5325838; doi:10.1007/s10875-016-0365-3)
Supplement: Supplementary file 3 — Gating strategy for the IL22-producig lymphocytes identification. (DOCX 62 kb) [file 10875_2016_365_MOESM3_ESM.docx]

**Supplementary Figure 2: Gating strategy for the IL22-producig lymphocytes identification.**


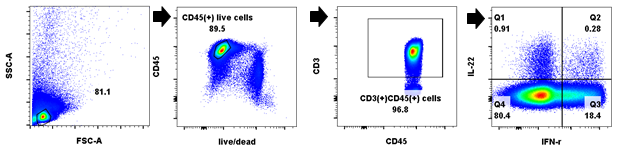


**Supplementary methods: LPMC and PBMC isolation.** PBMC was isolated by Ficoll density gradient centrifugation in SepMate^TM^ tubes. LPMC was isolated as previously described. Briefly, tissue was cut into small strips, minced by a sterile surgical scalpel, and incubated with 25ml digestion buffer (DMEM without sodium pyruvate, supplemented with 75U/ml Collagenase type XI, 20µg/ml Dispase neutral protease II, 500U/ml DNase, 0.5mM DTT and 1% FBS) at 37°C in the shaker for 30min at a constant speed of 175 rpm, filtered through a 70µm cell strainer. All cells were slowly cryopreserved in cell freezing medium and transferred to liquid nitrogen.
